# Molecular transformation of dissolved organic matter in paddy soils driven by electroactive microorganisms

**DOI:** 10.3389/fmicb.2026.1931831

**Published:** 2026-08-17

**Authors:** Xiaoshan Luo, Xun Wang, Qingyou Liu, Yin Ye, Lingyan Huang, Yong Yuan, Rong Tang

**Affiliations:** 1 School of Materials and Environmental Engineering, Shenzhen Polytechnic University, Shenzhen, China; 2 College of Ecology and Resources Engineering, Wuyi University, Wuyishan, China; 3 Guangdong Key Laboratory of Environmental Catalysis and Health Risk Control, Guangzhou Key Laboratory Environmental Catalysis and Pollution Control, School of Environmental Science and Engineering, Institute of Environmental Health and Pollution Control, Guangdong University of Technology, Guangzhou, China

**Keywords:** bioelectrochemical system, dissolved organic matter, electroactive microorganisms, molecular transformation, paddy soil

## Abstract

Electroactive microorganisms play important roles in extracellular electron transfer and soil carbon cycling. However, their effects on the molecular transformation of dissolved organic matter (DOM) in paddy soils remain poorly understood. Here, a bioelectrochemical system (BES) was used to enrich electroactive microorganisms from paddy soil, and DOM molecular transformation together with microbial community dynamics was characterized by ultrahigh-resolution Orbitrap mass spectrometry, 16S rRNA gene amplicon sequencing, and co-occurrence network analysis. Compared with the open-circuit control, enrichment of electroactive microorganisms reduced the aromaticity of soil DOM and promoted the relative accumulation of aliphatic compounds. As current generation reached a stable stage, the relative abundances of unsaturated hydrocarbons and condensed aromatic compounds decreased, whereas those of protein-/aliphatic-like compounds, carbohydrates, and lignin/carboxyl-rich alicyclic molecules increased, indicating substantial molecular transformation of soil DOM during extracellular electron transfer. Electroactive microorganisms, together with hydrocarbon- and polycyclic aromatic hydrocarbon-degrading bacteria, were significantly enriched during BES cultivation. Co-occurrence network analysis further revealed that DOM transformation was not driven solely by electroactive microorganisms but was achieved through their synergistic interactions with other functional microorganisms, facilitating the transformation of structurally complex DOM into more bioavailable low-molecular-weight compounds. These findings provide new insights into the microbial mechanisms underlying DOM transformation during extracellular electron transfer and improve our understanding of soil organic carbon cycling in paddy ecosystems.

## 1 Introduction

Electroactive microorganisms (EAMs) are capable of transferring electrons generated from the oxidation of organic substrates to extracellular electron acceptors via extracellular electron transfer (EET), thereby coupling organic matter degradation with electricity generation. Owing to this unique metabolic capability, bioelectrochemical systems (BESs) have been widely applied in energy recovery and the treatment of organic wastes and wastewaters (Chen et al., 2016; Rozendal et al., 2008). Recent studies have demonstrated that EAMs rarely function independently; instead, they establish syntrophic metabolic networks with other functional microorganisms, collectively driving organic matter transformation and electron fluxes within microbial communities (Yu et al., 2026). For example, electroactive bacteria can form cooperative metabolic relationships with phenol-degrading microorganisms, thereby enhancing the degradation of organic pollutants (Shi et al., 2021; Yang et al., 2025). Similarly, a mutualistic association between members of the Geobacteraceae and Desulfobulbaceae has been shown to contribute positively to current generation in bioelectrochemical systems (Ishii et al., 2013). In addition, *Geobacter* species can cooperate with fermentative microorganisms by utilizing acetate produced during fermentation for electricity generation (Rotaru et al., 2018). To date, electroactive microbial communities have been successfully enriched from a variety of environments, including marine sediments, activated sludge, and paddy soils (Dong et al., 2024; Joicy et al., 2019; Tang et al., 2024). Given their close involvement in substrate utilization and organic matter oxidation, the ecological roles of EAMs in soil carbon cycling have attracted increasing attention.

Dissolved organic matter is an important component of the active soil organic carbon pool and is primarily derived from the decomposition of plant residues, animal remains, and microbial metabolites (Li et al., 2022; Long et al., 2026). As a major source of carbon and energy for soil microorganisms, DOM plays a crucial role in nutrient cycling, organic matter transformation, and the maintenance of ecosystem functions in soils (Liu et al., 2023). The molecular composition and structural characteristics of DOM directly reflect the pathways and turnover dynamics of soil organic carbon; therefore, characterizing DOM molecular transformations is essential for understanding soil carbon cycling processes. In flooded paddy soils, DOM serves not only as an important substrate for microbial metabolism but also as a key intermediary linking organic matter decomposition, greenhouse gas production, and carbon sequestration. Consequently, changes in DOM composition can sensitively reflect microbial activities involved in soil carbon transformation processes. In recent years, advances in ultrahigh-resolution mass spectrometry (UHRMS) have provided powerful tools for the detailed characterization of complex DOM molecules and have been widely applied to investigate DOM sources, molecular composition, and transformation processes in natural environments (Leyva et al., 2023). Previous studies have demonstrated that the integration of spectroscopic techniques with UHRMS enables comprehensive characterization of DOM molecular features and their evolution across diverse environmental systems (Herzsprung et al., 2023; Zhang et al., 2021). Given that EAMs continuously oxidize organic substrates to sustain extracellular electron transfer, their metabolic activities are closely linked to the degradation and transformation of DOM. Moreover, extracellular electron transfer may alter microbial utilization strategies toward different DOM components, thereby influencing the enrichment, degradation, and transformation of specific molecular groups within the DOM pool. Despite these advances, whether EAMs actively participate in the transformation of soil DOM and how they influence DOM molecular composition and diversity remain largely unknown.

To fill these gaps, a bioelectrochemical system (BES) with a poised anode at +0.2 V as the extracellular electron acceptor was constructed to enrich electroactive microbial communities from paddy soil. By integrating 16S rRNA gene high-throughput sequencing, co-occurrence network analysis, and Orbitrap ultrahigh-resolution mass spectrometry, this study systematically investigated the effects of EAMs on the molecular composition and diversity of DOM in paddy soils. The specific objectives were to: (1) characterize the changes in the molecular composition and diversity of DOM under the influence of EAMs; and (2) reveal the associations between electroactive microbial communities and DOM molecules, and identify the key microbial taxa involved in DOM transformation. This study provides new insights into the interactions between electroactive microbial communities and DOM molecules, thereby advancing our understanding of soil organic carbon transformation processes in paddy soils.

## 2 Materials and methods

### 2.1 Collection and treatment of soil samples

Paddy soil samples were collected from a flooded rice field located in Dongguan City, Guangdong Province, a major rice-producing region in southeastern China (113.88° E, 23.06° N). Soil samples were obtained from three soil cores collected within an area of approximately 4 m^2^, with a sampling depth of 0–20 cm. After collection, the soil samples were transported to the laboratory and air-dried at room temperature in a well-ventilated and shaded environment. The dried samples were thoroughly homogenized and sieved through a 2-mm mesh to remove coarse debris and stones. The processed soil samples were subsequently stored for further experiments.

### 2.2 Construction and operation of soil-based bioelectrochemical systems (BES)

To evaluate the extracellular respiration capacity of soil, a three-electrode single-chamber soil BES reactor was constructed. The reactor consisted of a 100 mL cylindrical glass vessel with a lid, in which a sterilized graphite plate (1.5 × 2.0 × 0.5 cm), a titanium wire, and a saturated calomel electrode (SCE) were used as the working electrode, counter electrode, and reference electrode, respectively. Each reactor was filled with 40 g of soil and 2.0 cm of overlying water. The working electrode was horizontally embedded 1.0 cm below the soil-water interface, while the reference and counter electrodes were positioned in the water phase with their bottoms fixed 1.0 cm above the soil-water interface. Current generation was continuously monitored using a multichannel potentiostat (CHI 1000C, CH Instruments, Shanghai, China). All reactors were operated in the dark at 30 °C. Six closed-circuit (CC) reactors were established, with the working electrode poised at +0.2 V versus SCE to stimulate microbial extracellular respiration and characterize electroactive microorganism-driven DOM transformation. In parallel, six open-circuit (OC) reactors without an applied potential were set up as controls to represent the natural attenuation of DOM in soil. In addition, a portion of the original soil sample was retained and designated as “*Soil*” for subsequent analyses. When the current reached its peak value, three CC reactors and three OC reactors were dismantled and designated as *CCT* and *OCT*, respectively. When the current declined to a stable level (i.e., the current density in most reactors was lower than 20% of the maximum value), the remaining reactors were dismantled and designated as *CCL* and *OCL*, respectively. The cumulative charge output (*C*_*out*_), representing the total amount of electrons transferred from substrates to the electrode during current generation, was calculated by integrating the current–time (I-t) curve:


$$C_{o\,u\,t}=\int_{0}^{t}I\,dt$$

where I represents the current (A) and t represents the elapsed time (s).

### 2.3 DOM extraction and spectroscopic characterization

Dissolved organic matter was extracted from paddy soil samples (*Soil*, *OCT*, *CCT*, *OCL*, and *CCL*) collected at the end of reactor operation. Briefly, 4 g of soil was mixed with 20 mL of ultrapure water (1:5, w/v) in a polypropylene centrifuge tube and shaken at 170 rpm and 25 °C for 8 h, followed by centrifugation at 4000 rpm for 20 min (Li et al., 2018). The supernatant was filtered through a 0.45 μm mixed cellulose ester membrane and used for subsequent spectroscopic and mass spectrometric analyses. Dissolved organic carbon (DOC) concentrations were determined using a TOC analyzer (TOC-L CPH, Shimadzu, Japan). Prior to spectroscopic analyses, all samples were diluted to 10 mg C L^–1^ to minimize inner-filter effects. UV-Vis spectra were measured using a UV-2600 spectrophotometer (Shimadzu, Japan) over a wavelength range of 200–800 nm with ultrapure water as the blank. Absorption coefficients (a254, a280, and a354), specific ultraviolet absorbance (SUVA254, SUVA280, and SUVA350), and the absorbance ratio (E2/E3) were subsequently calculated (Jamieson et al., 2014). Excitation–emission matrix (EEM) fluorescence spectra were acquired using a fluorescence spectrophotometer (F-7000, Hitachi, Japan) with excitation wavelengths ranging from 200 to 500 nm and emission wavelengths ranging from 250 to 600 nm (Gueguen and Cuss, 2011). Fluorescence indices, including FI, BIX, and HIX, were calculated to characterize DOM properties (Kim et al., 2023). Detailed descriptions of the calculated spectral parameters are provided in Supplementary Text 1 and Supplementary Table 1.

### 2.4 DOM Orbitrap mass spectrometric analysis and molecular formula assignment

Collected DOM samples were pretreated using Bond Elut PPL solid-phase extraction cartridges (500 mg, 6 mL; Agilent Technologies). The cartridges were conditioned with 20 mL of HPLC-grade methanol followed by 20 mL of ultrapure water acidified to pH 2. Pre-filtered DOM samples acidified to pH 2 were then loaded onto the cartridges for solid-phase extraction, rinsed with 20 mL of pH 2 ultrapure water, and dried under a stream of high-purity N2. The extracted DOM was collected in 5 mL amber HPLC vials and stored at 4 °C in the dark until analysis. DOM samples were analyzed using an Orbitrap mass spectrometer (Q Exactive, Thermo Fisher Scientific) equipped with an electrospray ionization source operated in negative ion mode. Detailed instrumental parameters are provided in Supplementary Table 2. Internal calibration was applied to ensure mass accuracy, and spectra were acquired over a mass range of 100–1200 m/z. Methanol blanks, PPL extraction blanks, and solvent blanks were analyzed to monitor potential contamination, and peaks detected in blank samples were removed from the DOM datasets. Only peaks with a signal-to-noise ratio (S/N) greater than 5 were retained for subsequent analyses. Molecular formula assignment was performed using a previously reported MATLAB algorithm (Fu et al., 2020). The identified molecular formulas mainly contained C, H, O, N, and S, whereas compounds containing P and Cl were excluded from further analysis due to their low abundance (Yuan et al., 2017). Relative abundances of molecular groups were calculated by normalizing the signal intensity of each molecular formula to the total signal intensity. Based on the Van Krevelen diagram, the identified compounds were classified into seven categories, including lipids, proteins/aliphatics, lignins/cycloaliphatics, carbohydrates, unsaturated hydrocarbons, aromatics, and tannins, according to previously established classification criteria (Ohno et al., 2010; Smith et al., 2013). The stoichiometric boundaries used for compound classification are described in detail in the Supplementary Text 2.

### 2.5 DNA extraction and amplicon sequencing

Paddy soil samples collected from the *Soil*, *OCT*, *CCT*, *OCL*, and *CCL* treatments were subjected to bacterial community analysis. Total genomic DNA was extracted from 0.50 g of homogenized soil using the FastDNA SPIN Kit for Soil (MP Biomedicals, Santa Ana, CA, USA) according to the manufacturer’s instructions. The bacterial 16S rRNA gene was amplified using the universal primer pair 338F (5’-ACTCCTACGGGAGGCAGCAG-3’) and 806R (5’-GGACTACHVGGGTWTCTAAT-3’). PCR amplification was performed in a reaction mixture containing 10–100 ng of template DNA, 1 μL of each primer, and 25 μL of rTaq PCR premix. The purified PCR products were sequenced on an Illumina MiSeq platform by Majorbio Bio-Pharm Technology Co., Ltd., (Shanghai, China). Raw sequencing reads were processed using the Quantitative Insights Into Microbial Ecology (QIIME, v1.6.0) pipeline for quality filtering, sequence assembly, and operational taxonomic unit (OTU) clustering. Alpha diversity indices were calculated using Mothur (v1.30.2), with the Chao1 index used to estimate community richness and the Shannon index used to assess community diversity. The resulting data were subsequently used to characterize bacterial community composition and diversity across different treatments.

### 2.6 Co-occurrence network analysis

Co-occurrence network analysis was performed to explore the potential associations between DOM composition and microbial communities during electroactive enrichment. DOM composition was represented by the relative peak intensities of individual m/z ions, whereas microbial communities were represented by the relative abundances of bacterial genera (Luo et al., 2007). Spearman’s rank correlation coefficients between DOM molecules and bacterial genera were calculated using the “Hmisc” package (v4.1.2) in R. To reduce the number of pairwise comparisons and minimize network complexity, only the top 30 most abundant genera were included in the network analysis. Significant associations were identified based on both statistical significance (*P* ≤ 0.05) and correlation strength (|r| ≥ 0.90) (Che et al., 2021). In the resulting network, bacterial genera and m/z ions were represented as nodes, and significant correlations between nodes were represented as edges. Network visualization was performed using Gephi v0.9.2. Topological properties, including network diameter, average degree, clustering coefficient, and modularity, were calculated to characterize the network structure. Key microbial taxa were identified based on network topological metrics, including high degree, high closeness centrality, and low betweenness centrality.

## 3 Results and discussion

### 3.1 Variations in chemical and optical properties of DOM

**FIGURE 1:**
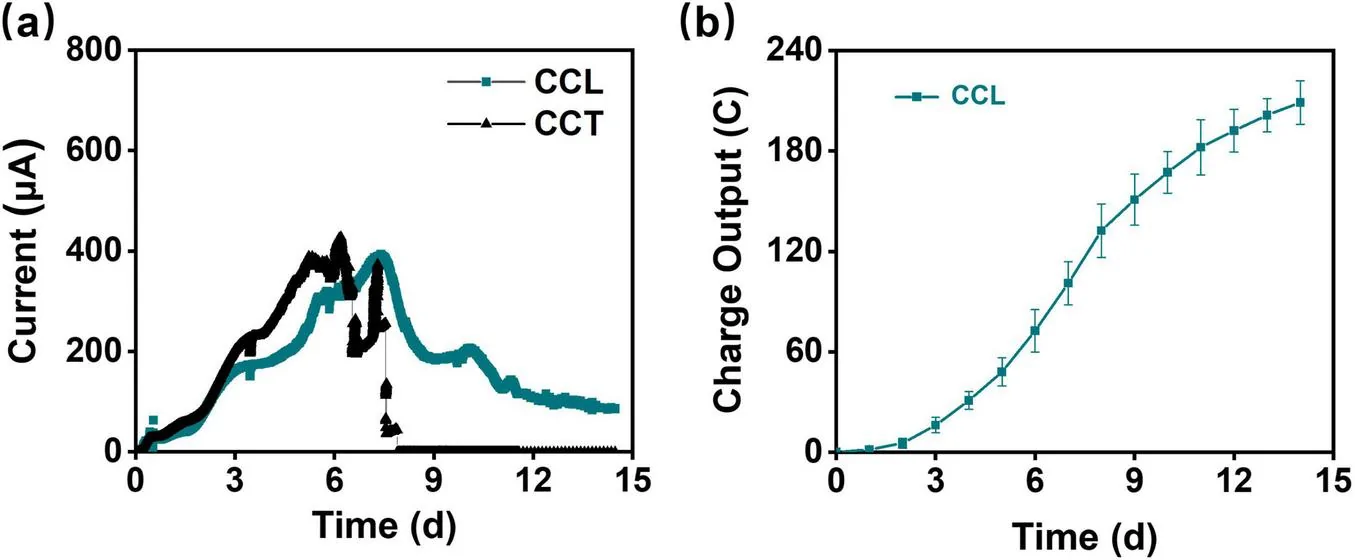
**(a)** Biocurrent and **(b)** charge output of a microbial electrochemical system.

The electron production by electroactive microorganisms in soil can be evaluated by monitoring the current generation of the BES. As shown in Figure 1a, the electroactive activity of microorganisms in the soil was stimulated along with the current generation. The generated current increased gradually and reached a maximum value of 416 μA. As presented in Figure 1b, the total coulombs (C) generated during the 14-day incubation period reached 208.9 °C. The chemical properties of DOM were analyzed for the experimental groups at the peak current stage (*CCT*) and stable current stage (*CCL*), their corresponding control groups (*OCT* and *OCL*), as well as (*Soil*. The results indicated that the total organic carbon (TOC) content of the samples exhibited a trend of increasing first and then decreasing over different periods. Notably, as shown in Table 1, the electroactive microbial community induced a sharp increase in TOC content at the peak current stage. The TOC concentration of the *CCT* soil sample was 39.52 ± 1.24 mg C/L, which was 1.4 times higher than that of the original soil sample (27.46 ± 1.12 mg C/L). This phenomenon suggested that the enriched electroactive bacteria participated in the oxidation of macromolecular insoluble organic matter, and the soluble organic carbon produced during metabolism triggered a significant increase in TOC (Wang et al., 2022). The oxidation of organic matter was accompanied by the generation of a large number of electrons, which were subsequently captured by the electrode, thereby leading to the variation in current. After the current stabilized, the TOC concentration of the *CCL* sample decreased, implying that the soluble organic carbon was utilized by electroactive microorganisms for the conversion of chemical energy into electrical energy. In contrast, the TOC concentrations in the corresponding control groups (*OCT* and *OCL*) were both higher than those in the experimental groups. This result demonstrated that driven by the potential difference of the BES, the experimental groups with electroactive microorganisms had a higher utilization efficiency of soluble organic matter, which also indirectly confirmed the enrichment of electroactive microorganisms in the experimental groups. In addition, the total nitrogen (TN) concentration increased continuously with the incubation time. After the current stabilized, the TN concentration of the *CCL* sample (58.56 ± 2.31 mg/L) was higher than that of the *OCL* sample (54.8 ± 2.05 mg/L). It could be inferred that the reduction process of nitrate might be significantly inhibited under the competition of the electrode as a dominant electron acceptor (Cao et al., 2025). Given that denitrification serves as the dominant pathway for nitrate reduction in anaerobic environments such as paddy soils, it could be further deduced that the electron competition between the electrode and nitrate was likely to result in an increase in the TN concentration within the system, which in turn led to the higher TN concentration in the experimental group *CCL* compared with its corresponding control group *OCL*.

**TABLE 1:** Carbon and nitrogen contents of DOM.

Samples	TOC (mgC/L)	TC (mgC/L)	IC (mgC/L)	TN (mgN/L)
Soil	27.46 ± 1.12	35.28 ± 1.33	7.83 ± 0.16	12.29 ± 0.45
OCT	68.96 ± 1.78	92.54 ± 3.36	23.58 ± 1.09	51.02 ± 2.02
CCT	39.52 ± 1.24	68.94 ± 2.11	29.42 ± 1.20	48.46 ± 1.97
OCL	34.86 ± 1.27	64.86 ± 2.83	30.02 ± 0.98	54.80 ± 2.05
CCL	33.26 ± 1.35	68.22 ± 2.67	34.96 ± 1.14	58.56 ± 2.31

The optical properties of DOM in soil samples collected at different cultivation stages were characterized using UV-Vis absorption spectroscopy and excitation–emission matrix (EEM) fluorescence spectroscopy. As shown in Supplementary Figure 1, weak absorption peaks at 280 nm were observed in the electroactive enrichment groups (*CCT* and *CCL*), suggesting the formation of carbonyl-containing compounds in the DOM as a result of electroactive microbial activity (Wang et al., 2026). In addition, the SUVA254 values of the control group (*OCL*) and the electroactive enrichment group (*CCL*) were 1.48 ± 0.11 and 1.31 ± 0.09, respectively. SUVA254 is generally positively correlated with the aromaticity and humification degree of DOM (Tang et al., 2025). The lower SUVA254 value in the electroactive enrichment group indicates that EAMs promoted the transformation of DOM components, resulting in a reduction in the overall aromaticity and humification degree of DOM. EEM fluorescence spectroscopy further revealed the variation characteristics of DOM molecules in soil samples from different groups during different incubation stages (Supplementary Figure 2 and Supplementary Table 3). At the peak current stage, the BIX values of DOM in the corresponding treatment groups (*OCT* and *CCT*) increased, reaching 0.84 ± 0.03 and 0.77 ± 0.01, respectively; whereas during the stable current stage, the BIX values in the *OCL* and *CCL* groups both decreased to 0.65. BIX is commonly used to indicate the relative contribution of freshly produced microbially derived organic matter in DOM. These results suggest that microbial metabolic activity was enhanced during the peak current stage, and the production of newly formed microbial DOM may have increased. Compared with the control group, the experimental groups showed slightly lower overall BIX values, indicating that electroactive microorganisms may further utilize newly produced microbial DOM and partially convert it into electrical energy through extracellular electron transfer processes (Wang et al., 2021). In addition, the HIX value of the *CCL* group was higher than that of *Soil*, indicating that the electroactive cultivation process may have promoted an increase in the degree of DOM humification. Meanwhile, changes in FI values further reflected alterations in DOM sources and composition. An increase in humification degree is generally beneficial for the stable accumulation of soil organic carbon and has potential positive implications for maintaining soil fertility and ecosystem functions. Overall, the combined UV-Vis and EEM spectroscopic analyses indicate that enrichment of electroactive microorganisms significantly altered the composition and optical properties of soil DOM, suggesting that they may play an important regulatory role in DOM molecular transformation processes.

### 3.2 Changes in DOM molecular composition driven by electroactive microorganisms

**FIGURE 2:**
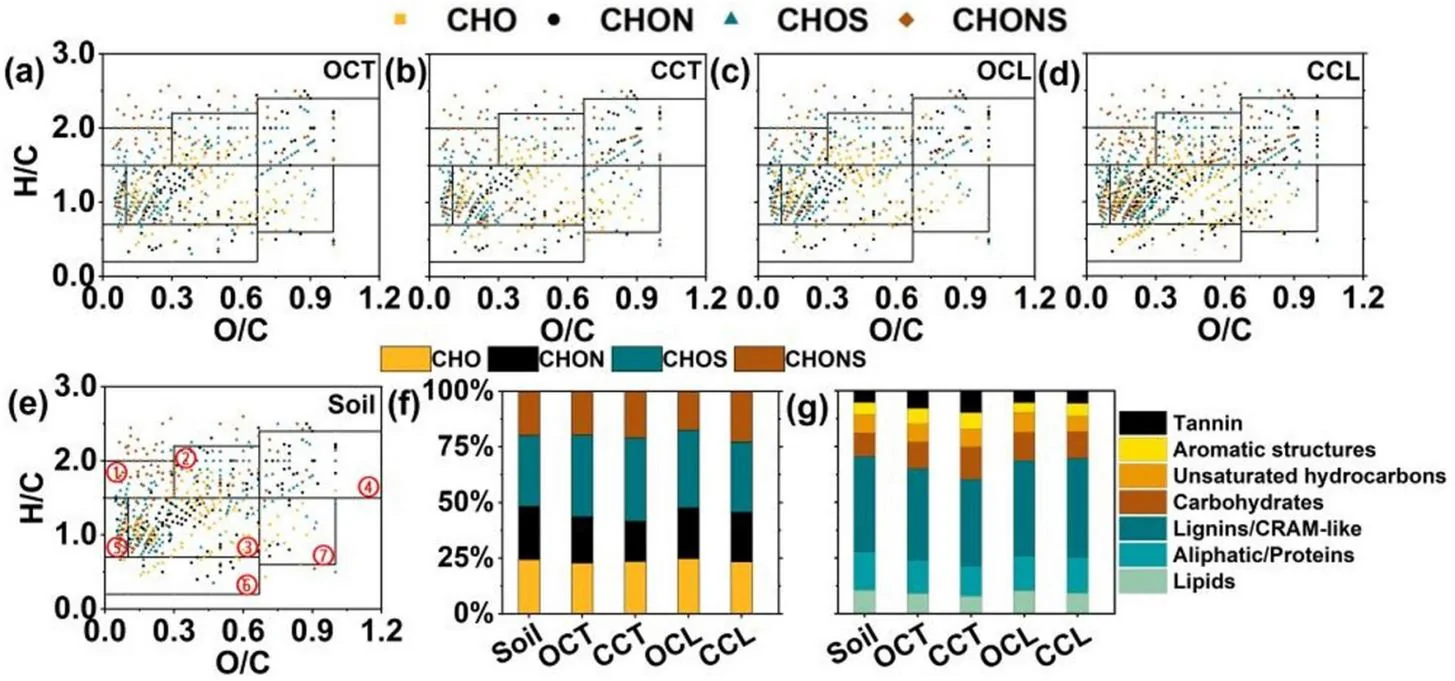
Changes of DOM components in Soil, OCT, CCT, OCL, and CCL samples during culture. **(a–e)** Van Krevelen diagrams of four major subclasses at different stages: CHO, CHON, CHOS, and CHONS. The numbers in the crossed lines represent different categories of recognized compounds in the DOM: 1: lipids; 2: aliphatic/protein; 3: Lignin/alicyclic rich molecules (similar to CRAM); 4: carbohydrates; 5: unsaturated hydrocarbons; 6: condensed aromatic; 7: Tannins; **(f)** The bar chart shows the percentages of the four main subcategories at different stages; **(g)** bar charts show the main compounds at different stages.

When the current reached its peak or entered the stable stage, significant changes were observed in a series of molecular parameters of DOM, including MW_*wa*_, O/C_*wa*_, N/C_*wa*_, S/C_*wa*_, H/C_*wa*_, AImod._*wa*_, DBE_*wa*_, NOSC_*wa*_, and DBE/C_*wa*_ (Supplementary Text 3). As shown in Supplementary Table 4, at the current peak stage, the DBE_*wa*_ and AImod._*wa*_ values of DOM in the CCT soil samples were 6.32 ± 0.48 and 0.37 ± 0.02, respectively, both of which were significantly higher than those in the CCL group at the current stable stage (5.55 ± 0.63 and 0.25 ± 0.01). This indicates that with prolonged enrichment of electroactive microorganisms, the aromaticity of soil DOM exhibited a trend of initial enhancement followed by subsequent decline (Zhang et al., 2019; Zheng et al., 2019). Meanwhile, the increase in H/C_*wa*_ further supports this result; the higher H/C_*wa*_ values also suggest that, at the current peak stage, the enrichment of electroactive microorganisms promoted the formation of aliphatic compounds in soil DOM, which is consistent with the variation trend of SUVA_254_ in the UV-Vis spectra. As shown in Figures 2a–e, Van Krevelen diagrams further demonstrate that, compared with the original soil and control groups, the number of DOM molecules in the experimental groups favoring the enrichment of electroactive microorganisms changed markedly, indicating that microbial enrichment promoted molecular transformation of DOM. Compared with *Soil*, when the enrichment cultivation of electroactive microorganisms reached the current stable stage, the number of DOM molecules in the experimental group *CCL* increased from 535 ± 32 to 750 ± 36. In contrast, in the control group *OCL*, where electroactive microorganisms were enriched without external voltage, the number of DOM molecules decreased from 535 ± 32 to 504 ± 25. These results suggest that the enrichment system of electroactive microorganisms may facilitate an increase in the chemical diversity and molecular richness of DOM. Notably, compared with Soil, at the current peak stage, the number of DOM molecules in the *CCT* and *OCT* groups decreased from 535 ± 32 to 465 ± 28 and 478 ± 21, respectively, indicating that microbial metabolic activity was highly active at this stage, and soluble organic matter represented by DOM was rapidly consumed. Meanwhile, the lower number of DOM molecules in *CCT* compared with *OCT* suggests that under externally applied voltage, the electroactive microbial enrichment system exhibited a higher DOM consumption rate (Maqbool and Jiang, 2023). With prolonged enrichment of electroactive microorganisms, the main molecular composition of DOM in paddy soil also underwent significant changes.

The distribution characteristics of four major DOM subcategories (CHO, CHON, CHOS, and CHONS) are shown in Figure 2f. The relative abundance of each fraction follows the order: CHOS compounds show the highest proportion (31.6%–37.4%), followed by CHO compounds (22.9%–24.8%), CHON compounds (18.0%–23.8%), and CHONS compounds (17.5%–22.7%). Notably, when the current reached its maximum, the CHOS content in the experimental group *CCT* reached 37.4%, approximately 1.2 times that of *Soil* (31.9%). The increase in CHOS and CHONS fractions indicates strong microbial activity, promoting the formation of related metabolic byproducts, and also reflects an increased degree of humification (Yu et al., 2019), which is consistent with the trend of the HIX index in the EEM results. Meanwhile, compared with the control group *OCT* (20.8%), the CHON content in *CCT* significantly decreased to 18.0%, indicating a marked reduction in amino acids and protein-like substances during the enrichment of electroactive microorganisms (Yan et al., 2026). This process was also accompanied by a decrease in CHO components representing carbohydrate-like substances (Zheng et al., 2024). These results suggest that during the current-peak stage in *CCT*, enhanced microbial metabolic activity may have led to increased biomass and promoted rapid transformation of DOM components, indicating that the electroactive microbial enrichment system facilitates the reconfiguration of soil DOM chemical composition. Figure 2g further illustrates the variation trends of seven classes of DOM compounds. In the original soil (*Soil*), molecules were mainly distributed in regions of lipids, proteins/aliphatic compounds, unsaturated hydrocarbons, and lignin/phenolic compounds. In contrast, in the *CCL* group, molecules were more enriched in regions of lignin/phenolic compounds, carbohydrates, condensed aromatic compounds, and tannin-related compounds. At the current peak stage, compared with *Soil*, the proportions of lipids and proteins/aliphatic compounds in *CCT* decreased from 10.66% and 17.05% to 8.04% and 13.39%, respectively, and were also lower than those in *OCT* (9.21% and 14.91%). Meanwhile, the proportions of condensed aromatics, tannins, and carbohydrates in *CCT* increased from 5.43%, 5.23%, and 10.47% to 7.37%, 9.82%, and 14.51%, respectively, and were higher than those in *OCT* (7.02%, 7.89%, and 11.84%). After the current decreased and stabilized, compared with *Soil*, the proportion of unsaturated hydrocarbons in *CCL* decreased from 8.33% to 6.95%, and was significantly lower than that in *OCL* (9.05%). Meanwhile, in *CCL*, the proportions of lignin/phenolic compounds and carbohydrates increased from 42.83% and 10.47% to 44.41% and 11.85%, respectively, with lignin/phenolic compounds being higher than those in *OCL* (42.80%). Notably, at the current peak stage, the proportion of lignin/phenolic compounds in *CCT* sharply decreased to 38.84%, which was lower than both *Soil* (42.83%) and *CCL* (44.41%).

**FIGURE 3:**
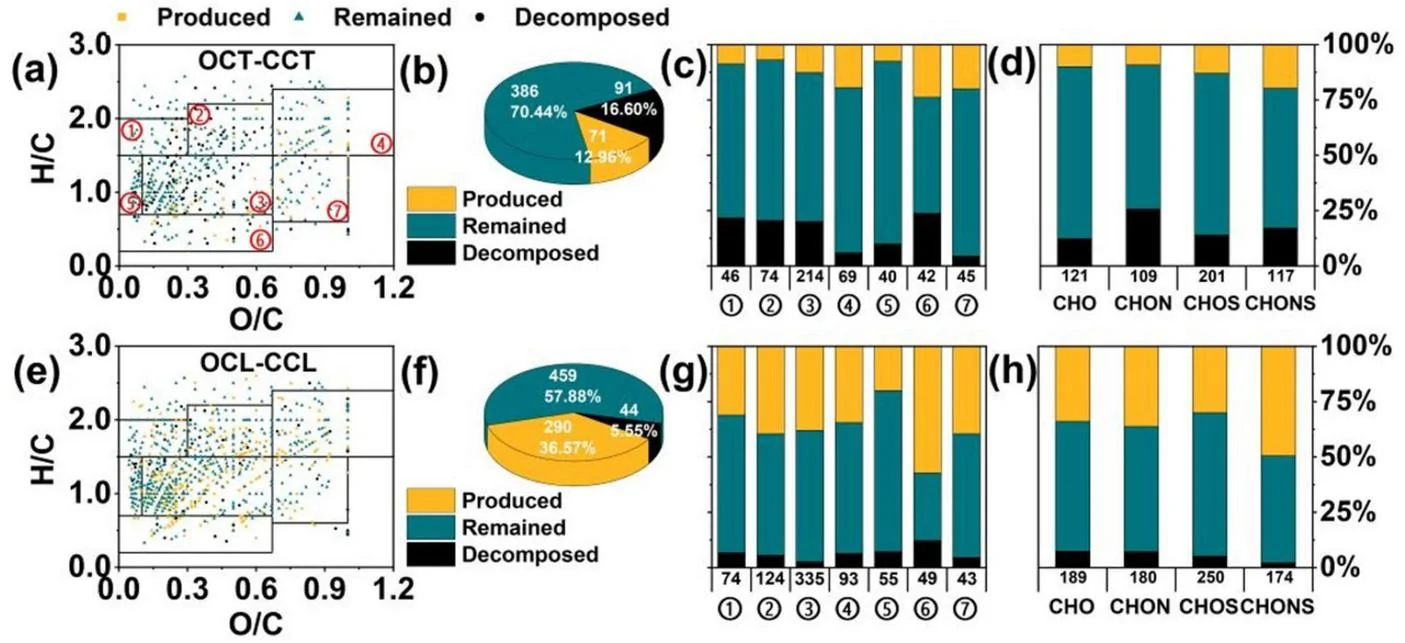
**(a,e)** Compare the Van Krevelen diagrams decomposed, retained, and generated in the different stages of the DOM. The numbers in the crossed lines represent the different biochemical classes in the DOM :1: lipids; 2: aliphatic/protein; 3: Lignin/carboxy-rich alicyclic molecules; 4: carbohydrates; 5: unsaturated hydrocarbon; 6: aromatic structure; 7: Tannic acid; **(b,f)** percentage of the comparator decomposed, retained, and generated in the DOM; **(c,g)** The bar chart shows the percentage of seven identified DOM compounds; **(d,h)** The bar chart shows the percentages of the four subcategories. The number of each identified DOM combination is shown below the *x* axis.

Through comparison of the presence and absence of DOM molecules in *OCT* vs. *CCT* and *OCL* vs. *CCL* samples, the molecules that were removed, persisted, or newly produced during DOM transformation under electroactive microorganism enrichment were further identified. As shown in Figures 3a,e, the distributions of degraded compounds, resistant compounds, and newly produced compounds in DOM were visualized using Van Krevelen diagrams. As shown in Figure 3b, at the peak current stage, compared with *OCT*, *CCT* contained 91 degraded molecules and 71 newly produced molecules. Figure 3c shows that the degraded molecules were mainly distributed in regions corresponding to lipids, proteins/aliphatics, lignin/cycloaliphatics, and condensed aromatics, whereas the newly produced molecules were mainly distributed in carbohydrate and tannin regions. In addition, Figure 3d indicates that CHON-containing molecules accounted for the largest proportion of degraded compounds, while CHONS-containing molecules accounted for the largest proportion of newly produced compounds. At the stable current stage, as shown in Figure 3f, compared with *OCL*, *CCL* contained 44 degraded molecules and 290 newly produced molecules. Figure 3g shows that the degraded molecules were mainly distributed in unsaturated hydrocarbons and condensed aromatic regions, whereas the newly produced molecules were mainly distributed in protein/aliphatic, carbohydrate, and lignin/cycloaliphatic regions. Moreover, Figure 3h indicates that CHONS-containing molecules still accounted for the largest proportion of newly produced compounds.

Previous studies have shown that the electroactive cultivation process can utilize energy from volatile fatty acids (VFAs) and, through coupling with VFA biodegradation, VFAs can serve as direct substrates for electroactive microorganisms (Logan et al., 2008). Therefore, both the peak current stage and the stable current stage exhibited significant degradation of lipids and protein/aliphatic compounds. At the peak current stage, CHON-containing molecules showed the highest degree of degradation, indicating a sharp decrease in amino acids and proteins, further supporting this interpretation. In addition, renewable organic substrates (e.g., lignocellulose-rich materials) possess high energy potential (Hassan et al., 2018), and their degradation typically involves the synergistic action of dark fermentation and electroactive processes, during which substrates are hydrolyzed and fermented into VFAs. VFAs can then be further utilized by electroactive microorganisms, enabling efficient electricity generation in BESs while simultaneously promoting lignin removal. Meanwhile, the rapid increase in VFAs also enhances microbial metabolic activity, leading to an increase in CHONS-containing compounds. The BES cultivation process can also lyse microbial cells trapped in solid sediments and release biopolymers (e.g., soluble proteins and carbohydrates) (Yin et al., 2019). Therefore, it promoted the accumulation of carbohydrates in *CCT* and *CCL*. In addition, studies have shown that the positive interaction between electroactive bacteria and phenol-degrading bacteria facilitates efficient phenol removal (Wang et al., 2019), thereby leading to a decrease in the proportion of unsaturated hydrocarbons. Overall, the experimental results demonstrate that BES operation was accompanied by pronounced changes in DOM molecular composition. The enrichment of electroactive microorganisms, together with previous knowledge of extracellular electron transfer processes, suggests that microbial electroactivity may contribute to these transformations. At the peak current stage, the main features are a decrease in lipids, proteins/aliphatics, and lignin/cycloaliphatics, along with an increase in tannins and carbohydrates. At the stable current stage, the main features are a decrease in unsaturated hydrocarbons and condensed aromatics, along with an increase in protein/aliphatics, carbohydrates, and lignin/cycloaliphatics. This process may facilitate the efficient conversion of chemical energy into electrical energy by electroactive microorganisms, while also promoting the accumulation of bioavailable small molecules (e.g., carbohydrates), thereby enhancing the overall humification level of the system.

### 3.3 Community structure of potential electroactive microorganisms in paddy soil

The α-diversity of microbial communities in the soils surrounding the BES working electrode at different cultivation stages is summarized in Supplementary Table 5. Compared with the control groups (*OCT* and *OCL*), which had Shannon indices of 6.82 ± 0.13 and 8.63 ± 0.56, respectively, the electroactive enrichment groups (*CCT* and *CCL*) exhibited higher microbial diversity (Shannon indices of 7.91 ± 0.28 and 8.71 ± 0.95, respectively), greater community richness (Chao1 index), and a larger number of OTUs. Microbial diversity is an important ecological indicator reflecting community structure, functional potential, and ecosystem stability. The OTU numbers in *OCT* (2438 ± 226) and *OCL* (2997 ± 278) were lower than those in *CCT* (2781 ± 251) and *CCL* (3070 ± 276), suggesting that the working electrode, serving as an extracellular electron acceptor, exerted a selective pressure on the microbial community. Through extracellular electron transfer and the oxidation of organic substrates, EAMs likely promoted the restructuring of the microbial community, resulting in increased richness and diversity. The enhanced microbial diversity may also contribute to more stable electricity generation (Xin and Qiu, 2021). Rarefaction curves for all samples are presented in Supplementary Figure 3a. As sequencing depth increased, all rarefaction curves gradually approached a plateau, indicating that the majority of OTUs had been captured and that the sequencing depth was sufficient to comprehensively characterize the microbial communities in the soil samples. The β-diversity analysis shown in Supplementary Figure 3b revealed greater differences in microbial community composition when the current reached its maximum, whereas the differences became less pronounced after the current stabilized. These results indicate that different cultivation stages exerted distinct effects on the structure of the soil microbial community.

**FIGURE 4:**
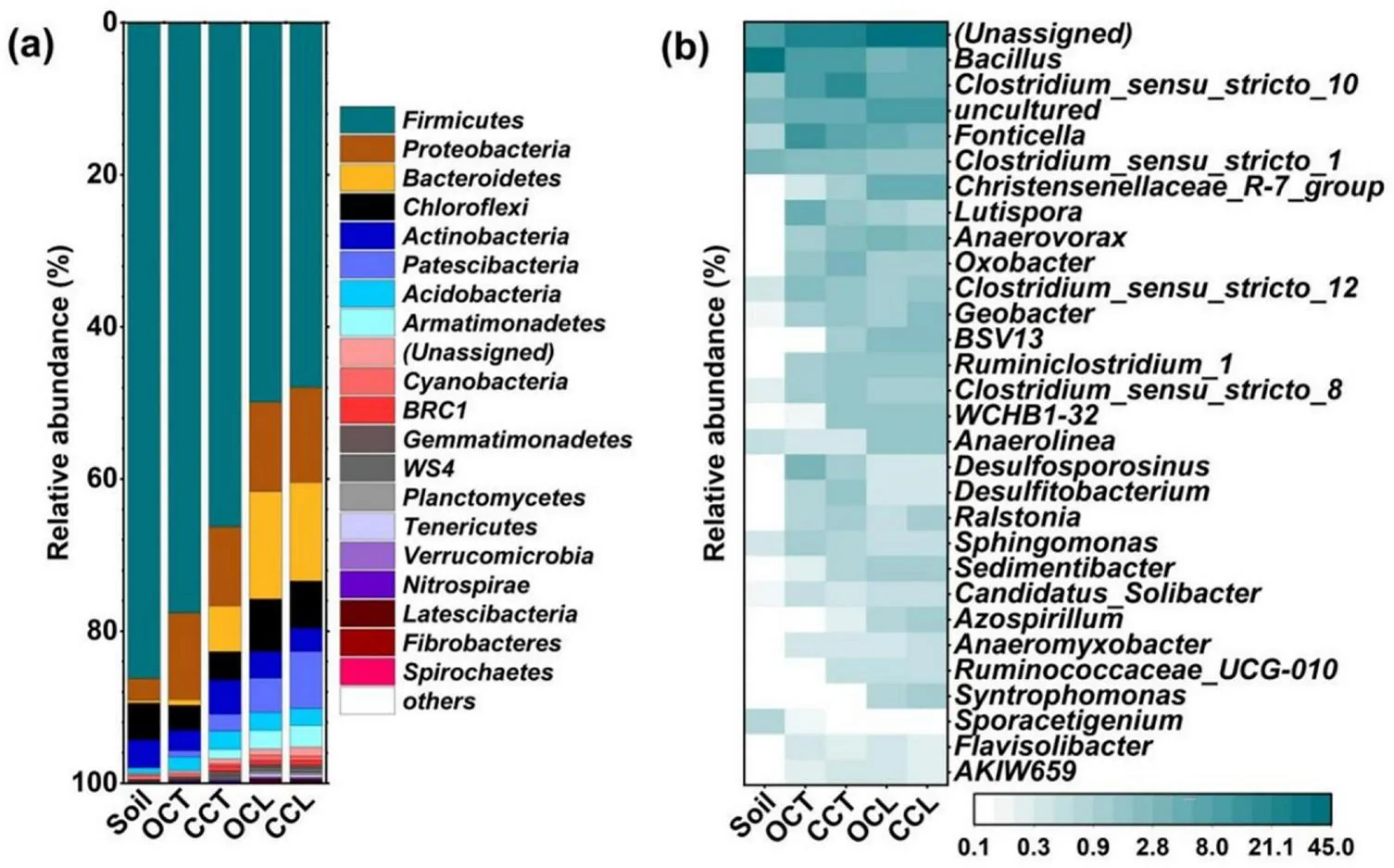
**(a)** Relative abundance of the top 20 bacterial taxa at phylum level at different culture stages; **(b)** relative abundances of the top 30 bacterial groups at the generic level at different culture stages.

To identify key electroactive microorganisms in soil, the microbial community structures in soils surrounding the working electrodes of microbial reactors at different stages were analyzed. As shown in Figure 4a, Firmicutes dominated all samples. At the phylum level, the main groups included Firmicutes (47.9%–86.2%), Proteobacteria (2.9%–12.5%), Bacteroidetes (0.4%–14.2%), Chloroflexi (3.2%–6.8%), Actinobacteria (2.7%–4.5%), Patescibacteria (0.1%–7.5%), Acidobacteria (0.7%–2.4%), and Armatimonadetes (0.1%–2.8%). The combined relative abundance of these dominant phyla exceeded 95% of total bacterial taxa, which is consistent with most BES-related studies (Thakur et al., 2026). Significant differences in microbial community structure were observed across different cultivation stages, indicating dynamic shifts in community composition over time. At the stage of maximum current, compared with the control group *OCT*, the relative abundances of Firmicutes and Proteobacteria in the experimental group *CCT* decreased markedly from 77.6% and 11.4% to 66.2% and 10.4%, respectively. Notably, the relative abundance of Proteobacteria in *CCT* was significantly higher than in Soil, being 3.6 times greater. Meanwhile, the relative abundances of Bacteroidetes, Chloroflexi, Actinobacteria, Patescibacteria, Acidobacteria, and Armatimonadetes increased substantially from 0.8%, 3.2%, 2.7%, 0.8%, 1.7%, and 0.3% to 6.0%, 3.7%, 4.5%, 2.2%, 2.4%, and 1.3%, respectively, corresponding to 7.9, 1.1, 1.7, 2.6, 1.4, and 4.9-fold increases relative to *OCT*. During the stage when current decreased and stabilized, compared with the control group *OCL*, the relative abundances of Firmicutes, Bacteroidetes, Chloroflexi, Actinobacteria, and Acidobacteria in the experimental group *CCL* decreased from 49.8%, 14.1%, 6.9%, 3.5%, and 2.4% to 47.9%, 12.9%, 6.1%, 3.0%, and 2.2%, respectively. In contrast, the relative abundances of Proteobacteria, Patescibacteria, and Armatimonadetes increased from 11.7%, 4.9%, and 2.5% to 12.5%, 7.5%, and 2.8%, respectively. Overall, compared with Soil and the *OCT* and *CCT* samples at peak current, the *OCL* and *CCL* samples at the stabilized current stage showed a decreasing trend in the relative abundance of Firmicutes, while Proteobacteria, Bacteroidetes, Chloroflexi, Patescibacteria, Acidobacteria, and Armatimonadetes showed increasing trends, with more pronounced changes in the experimental groups. This indicates that applied voltage selectively enriches specific microbial communities on the working electrodes.

Many studies have identified various electroactive microorganisms within the phyla Firmicutes, Proteobacteria, Actinobacteria, and Acidobacteria, particularly within *Alpha-, Beta-, Gamma-*, and Deltaproteobacteria, as well as certain Firmicutes and Acidobacteria lineages (Garbini et al., 2023; Jiang et al., 2016). As important potential electroactive taxa, the increased relative abundance of Proteobacteria, Actinobacteria, and Acidobacteria in the experimental groups may be associated with their enrichment under enhanced extracellular electron transfer processes. Some Firmicutes anaerobes have been shown to exhibit synergistic interactions with electroactive microorganisms during the degradation of complex organic matter (Liu et al., 2016). In addition, Bacteroidetes-dominated anodic biofilm communities have been reported to generate high current densities in thermophilic BES systems, suggesting that Bacteroidetes may play an important role in electricity generation in the *CCT* and *CCL* reactors. Chloroflexi are known to contribute to the degradation of carbohydrates and other cellular components such as amino acids in bioreactors, indicating their involvement in organic matter degradation and nitrogen removal processes (Bovio-Winkler et al., 2023), and are therefore commonly found in various wastewater treatment systems. Armatimonadetes possess the ability to utilize a wide range of carbohydrates and recalcitrant extracellular cellulose, and have also been implicated in anaerobic peptidoglycan degradation. In summary, with increasing cultivation time, microbial diversity and richness gradually increased, indicating that the enrichment of electroactive microorganisms drives the soil system toward selective enrichment and succession of dominant functional microbial groups.

As shown in Figure 4b, in the experimental groups *CCT* and *CCL*, a variety of electroactive microorganisms, including *Bacillus*, *Clostridium*_sensu_stricto_10, *Clostridium*_sensu_stricto_1, *Clostridium*_sensu_stricto_12, *Geobacter*, *Clostridium*_sensu_stricto_8, *Anaerolinea*, *Desulfitobacterium*, *Sedimentibacter*, *Azospirillum*, and *Syntrophomonas*, were significantly enriched compared with the control groups *Soil*, *OCT*, and *OCL*. At the stage of maximum current, the relative abundances of *Clostridium*_sensu_stricto_10, *Desulfitobacterium*, *Sedimentibacter*, *Azospirillum*, and *Syntrophomonas* in *CCT* were 1.7, 2.3, 2.9, 3.3, and 2.2-fold higher than those in *OCT*, respectively. During the stage when the current decreased and stabilized, the relative abundance of *Geobacter* in *CCL* was 1.8 times that in *OCL*. In addition, synergistic interactions among different microbial groups played an important role in both organic matter degradation and electricity generation. *Fonticella* promotes the degradation of complex organic matter, particularly proteins, enhances the production of VFAs, and generates acetate through hydrolytic acidification (LaBelle et al., 2020). Acetate serves as a key electron donor for most electroactive microorganisms; therefore, the presence of *Fonticella* is beneficial for their growth. Christensenellaceae plays a crucial role in the oxidation of volatile short-chain fatty acids, thereby optimizing the environment for hydrolytic acetate production (Li et al., 2020), and further contributing to the degradation of VFAs produced by *Fonticella* metabolism. *Lutispora* is involved in cellulose degradation and protein fermentation (Jiang et al., 2020; Song et al., 2025), showing functional similarity to *Fonticella*. *Anaerovorax* is capable of degrading proteins, carbohydrates, and cellulose (Kong et al., 2022). *Ruminiclostridium*_1 is known for degrading lignocellulose and producing ethanol. Electroactive microorganisms can utilize the degradation products from other microorganisms to generate electricity (Wang et al., 2023). For example, *Ruminiclostridium cellulolyticum* ferments cellulose, and its metabolic products can enhance current generation by *Geobacter sulfurreducens*. Most of the above genera exhibited higher relative abundances in *CCT* at the maximum current stage compared with *Soil* and *CCL*, which is consistent with the decreased proportions of lipids, proteins/aliphatic compounds, and lignin/phenolic compounds.

This indicates that the enrichment of electroactive microorganisms enhances the abundance of protein- and cellulose-degrading bacteria, thereby improving the utilization efficiency of these compounds. During different cultivation stages, *CCT* and *CCL* were also enriched with various organic matter-degrading functional groups. For example, *Oxobacter* promotes the degradation of compounds such as PCP and produces organic acids (Jia et al., 2026). *Desulfosporosinus* can biodegrade monoaromatic compounds such as toluene (Abu Laban et al., 2015). *Desulfitobacterium* plays an important role in the bioremediation and natural attenuation of chlorinated aliphatic and aromatic hydrocarbons (e.g., anisole and pentachlorophenol) (Futagami et al., 2013). *Ralstonia* exhibits high efficiency in degrading various organic pollutants and can degrade phthalates under anaerobic conditions (Georgoulis et al., 2021), and *Sphingomonas* is capable of degrading polycyclic aromatic hydrocarbons (Zhou et al., 2012). The enrichment of these microbial groups is closely associated with the degradation of unsaturated hydrocarbons and condensed aromatic compounds at different cultivation stages. Overall, synergistic interactions between electroactive microorganisms and other functional microbial groups promote the transformation of refractory and high-molecular-weight organic carbon in paddy soil DOM, consistent with the results of ultrahigh-resolution mass spectrometry. These findings suggest that in mixed-culture soil systems, microbial communities do not exhibit strong competitive exclusion but instead form cooperative metabolic networks, showing simultaneous potential for pollutant removal and bioelectricity generation. Electroactive microorganisms play a dominant role in structuring and coordinating these community interactions.

### 3.4 Role of potential electroactive microorganisms in DOM transformation

**FIGURE 5:**
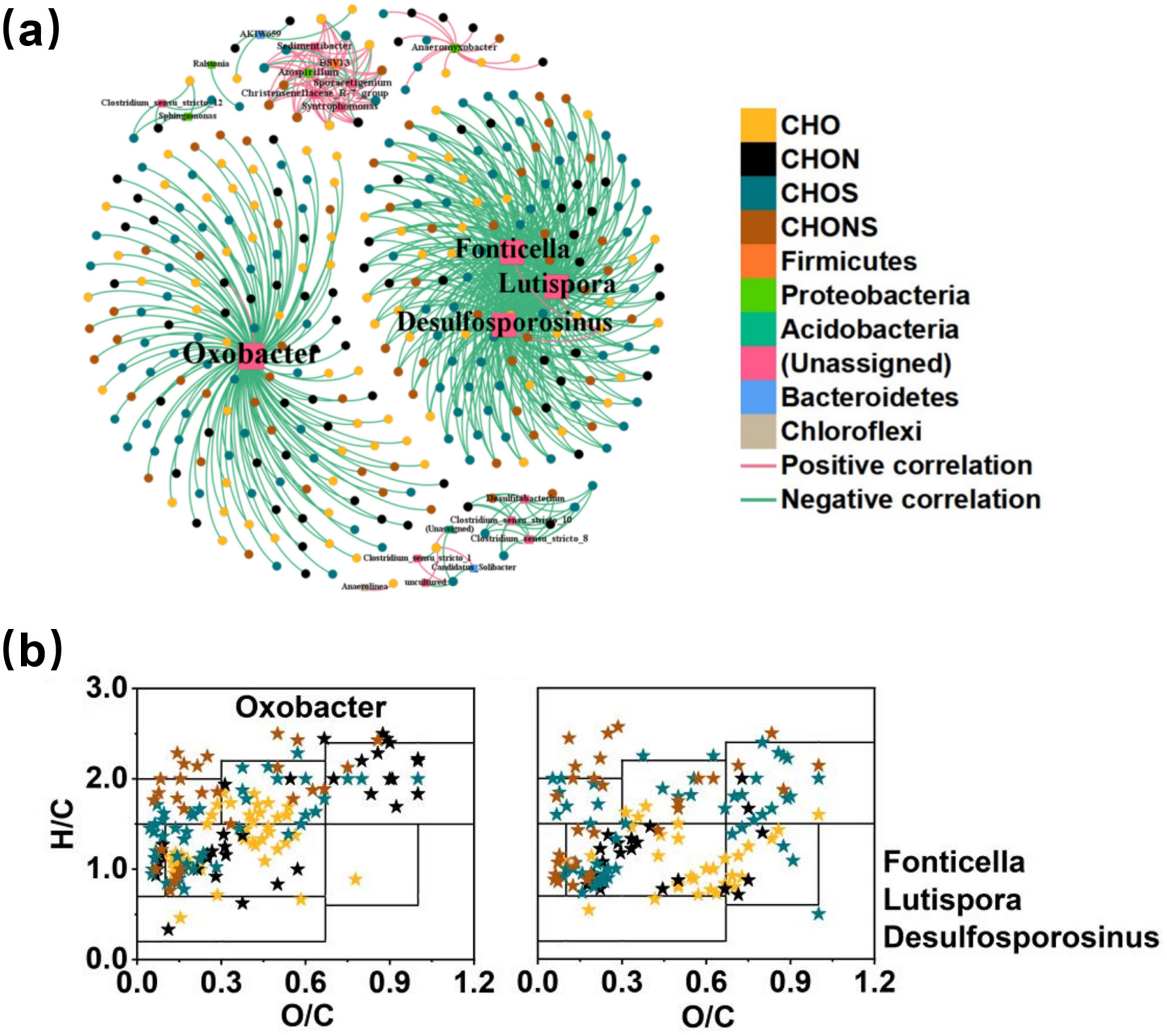
**(a)** Co-occurrence network illustrating the relationships between microbial genera and four DOM subclasses during BES enrichment. Square nodes represent microbial genera and are color-coded according to their phyla, whereas circular nodes represent the four DOM subclasses. The colors of the nodes and edges are explained in the legend. **(b)** Van Krevelen diagrams of the first four top microbial genera.

To investigate the roles of potential EAMs and other microbial taxa in DOM transformation, a co-occurrence network analysis was performed. As shown in Figure 5a, the co-occurrence network constructed between microbial communities and DOM molecules consisted of 353 nodes and 686 edges, with a modularity index of 0.59, indicating a well-defined modular structure. Microorganisms closely associated with DOM molecules were mainly affiliated with the phyla Firmicutes, Proteobacteria, Acidobacteria, Bacteroidetes, and Chloroflexi. This observation is consistent with the enrichment of potential EAMs in the BES, suggesting a close association between potential EAMs, their associated microbial communities, and DOM transformation. Each module may represent a relatively independent microbial assemblage involved in DOM transformation. Overall, negative correlations predominated between microorganisms and DOM molecules, indicating that most microorganisms participated in the utilization and degradation of DOM components. In contrast, only a few modules exhibited positive correlations, suggesting that these microorganisms may facilitate the production or accumulation of specific DOM components through their metabolic activities. As shown in Figure 5b, the largest module was dominated by *Oxobacter* and exhibited predominantly negative correlations with lipid-like, protein/aliphatic-like, unsaturated hydrocarbon-like, carbohydrate-like, and lignin/cycloaliphatic-like compounds. The second-largest module was primarily composed of *Fonticella*, *Lutispora*, and *Desulfosporosinus*, which were negatively correlated with lipid-like, protein/aliphatic-like, unsaturated hydrocarbon-like, carbohydrate-like, lignin/cycloaliphatic-like, and tannin-like compounds. Previous studies have demonstrated that *Fonticella* is capable of degrading complex organic matter, particularly proteins; *Lutispora* can decompose cellulose and utilize proteins; and *Desulfosporosinus* possesses the capacity to degrade aliphatic and aromatic compounds. Therefore, the observed negative correlations between these genera and specific DOM components suggest that they may contribute to the degradation and transformation of these organic compounds. The third module was mainly characterized by positive correlations among three potential EAMs and three other functional microorganisms, suggesting potential ecological associations or complementary metabolic functions. It is likely that metabolites produced by the associated microorganisms provide substrates or favorable growth conditions for potential EAMs, thereby promoting their growth and metabolic activity. These findings are consistent with the results obtained from ultrahigh-resolution mass spectrometry and microbial community analyses, further suggesting that DOM transformation is not mediated solely by potential EAMs but rather is achieved through coordinated metabolic interactions within a complex microbial network.

## 4 Conclusion

This study constructed a BES to enrich EAMs from paddy soil, enabling a systematic investigation of the relationships among DOM molecular composition, microbial community structure, and their interactions during electroactive enrichment. EAMs promoted the molecular transformation of DOM by decreasing the relative abundance of recalcitrant compounds, including aromatic compounds, unsaturated hydrocarbons, and condensed aromatic structures, while increasing the abundance of more biodegradable compounds such as carbohydrates and enhancing the humification degree of DOM. Meanwhile, electroactive enrichment reshaped the soil microbial community, increased microbial diversity, and enriched multiple functional taxa associated with organic matter degradation. Co-occurrence network analysis further demonstrated that DOM transformation was not mediated solely by potential EAMs but instead relied on a synergistic metabolic network formed between potential EAMs and other functional microorganisms, collectively driving the degradation and transformation of complex organic matter and promoting carbon cycling in paddy soils.

## Data Availability

The data presented in the study are deposited in the NCBI repository, accession number PRJNA1503187.
