# Supplementary material for: Molecular transformation of dissolved organic matter in paddy soils driven by electroactive microorganisms
Source: Front Microbiol. 2026 Aug 17;17:1931831. doi: 10.3389/fmicb.2026.1931831 (PMC13526929; doi:10.3389/fmicb.2026.1931831)
Supplement: Supplementary file 1 [file Supplementary_file_1.docx]

**Supplementary materials**

**Molecular transformation of dissolved organic matter in paddy soils driven by electroactive microorganisms**

Xiaoshan Luo^1^, Xun Wang ^1^, Qingyou Liu^1^, Yin Ye^2^, Lingyan Huang^2^, Yong Yuan^3*^, Rong Tang^2*^

^1^ School of Materials and Environmental Engineering, Shenzhen Polytechnic University, Shenzhen, 518055, China

^2^ College of Ecology and Resources Engineering, Wuyi University, Wuyishan 354300, China.

^3^ Guangdong Key Laboratory of Environmental Catalysis and Health Risk Control, Guangzhou Key Laboratory Environmental Catalysis and Pollution Control, School of Environmental Science and Engineering, Institute of Environmental Health and Pollution Control, Guangdong University of Technology, Guangzhou, 510006, China

* CORRESPONDENCE

Yong Yuan

[yuanyong@soil.gd.cn](mailto:yuanyong@soil.gd.cn)

Rong Tang

13060877725@163.com

**Text S1** Soil properties measurements

Soil total organic carbon (TOC) and total carbon (TC) were determined by SSM-5000A solid module of Total Organic Carbon Analyzer (TOC-L CPH, Shimadzu, Japan). Soil dissolved total nitrogen (TN) was extracted using 0.5 mol·L^-1^ K_2_SO_4_ at 1:5 (weight:volume) and measured using Total Organic Carbon Analyzer (TOC-L CPH, Shimadzu, Japan). All measurements were conducted in triplicate, and average value is presented.

**Text S2** Boundaries of regions in van Krevelen diagrams

In this study, we divided the van Krevelen diagrams into several regions corresponding to seven classes of compounds found in DOM, including lipids, aliphatic/proteins, lignins/carboxylic rich alicyclic molecules (CRAM)-like structures, carbohydrates, unsaturated hydrocarbons, aromatic structures and tannins, according to previously published reports. The stoichiometric ranges used to establish the boundaries of the classification are as follows:

lipids, H/C = 1.5- 2.0, O/C = 0- 0.3;

aliphatic/proteins, H/C = 1.5- 2.2, O/C = 0.3- 0.67;

lignins/CRAM-like structures, H/C = 0.7-1.5, O/C = 0.1-0.67;

carbohydrates, H/C = 1.5-2.4, O/C = 0.67-1.2;

unsaturated hydrocarbons, H/C = 0.7-1.5, O/C = 0-0.1;

aromatic structures, H/C = 0.2-0.7, O/C = 0-0.67;

and tannin, H/C = 0.6-1.5, O/C = 0.67-1.0.

**Text S3** Attributes of DOM molecules and samples

(1) Double bond equivalent (DBE)^1^:

$$\text{DBE=}\frac{\text{2 × C + N + P - H + 2}}{2}$$

DBE indicates the unsaturation of a specific molecule.

(2) Nominal oxidation state of carbon (NOSC)^2^:

$$\text{NOSC=4-}\frac{\text{4 × C + H - 2 × O - 3 × N - 2 × S + 5 × P}}{\text{C}}$$

(3) Modified aromaticity index (AI_mod_)^3^:

where in (1) (2) (3), C, H, O, N, S and P are the number of carbon, hydrogen, oxygen, nitrogen, sulfur, and phosphorus atoms in a given molecular formula.

**Table S1** Calculation of UV-Vis spectral parameters and fluorescence spectral parameters

| Index | Calculation | Description |
| --- | --- | --- |
| a_λ_ | a_λ_ = 2.303A_(λ)_/*l*, where a_λ_ (m^-1^) is Napierian absorption coefficient, A_(λ)_ is the absorbance at wavelength of λ nm and *l* is the path length (m). | Absorption coefficient at wavelength of λ nm. a_254_ and a_280_ indicate the concentration of aromatic fraction of DOM and a_350_ indicates the concentration of lignin fraction of DOM. |
| SUVA_λ_ | SUVA_λ_ = a_λ_/[DOC], where SUVA_λ_ (L mgC^-1^ m^-1^) is the DOC-normalized absorbance coefficient. [DOC] is dissolved organic carbon concentration. | DOC-normalized absorbance coefficient at wavelength of λ nm. SUVA_254_ and SUVA_280_ indicate the aromaticity of DOM and SUVA_350_ indicates the lignin content of DOM. |
| E_2_/E_3_ | E_2_/E_3_ = A_(250)_/A_(365)_, where E_2_/E_3_ is absorbance ratio, A_(250)_ and A_(365)_ were the absorbance at 250 nm and 365 nm, respectively. | Absorbance ratio E_2_/E_3_: indicates changes in the relative size of DOM molecules: higher E_2_/E_3_ indicates low molecular weight. |
| FI | FI = I_Ex370:Em470_ / I_Ex370: Em520_, where FI is the fluorescence index and I is the fluorescence intensity at certain excitation and emission wavelength. | Fluorescence index FI: identifies the relative contribution of terrestrial and microbial sources to the DOM pool. Higher FI values indicate more contribution of microbial origins, showing the dominant of terrestrial (< 1.4) and microbial (> 1.9) DOM. |
| BIX | BIX = I_Ex310:Em380_ / I_Ex310: Em430_, where BIX is the biological index and I is the fluorescence intensity at a certain excitation and emission wavelength. | Biological index BIX: reflects the autotrophic productivity and the relative contribution of DOM autochthonous source in water, showing very less ( < 0.6) and more ( > 1) autochthonous DOM. |
| HIX | HIX = Area _Ex254:Em435 - Em480_ / (Area _Ex254:Em435 - Em480_ + Area _Ex254:Em300 - Em345_), where HIX is the humification index and Area is the emission spectra area from the range of certain emission wavelength at a specific excitation wavelength. | Humification index HIX: indicates the content of humic substance or the extent of humification. Higher HIX values indicate an increasing degree of humification or abundance of humic substance. |

**Table S2** Q Exactive quad-electrostatic field track well high resolution mass spectrometry parameters

| Mass spectrum parameters | set value |
| --- | --- |
| Ion Source | H-ESI |
| Sheath gas flow rate | 7 |
| Aux gas flow rate | 0 |
| Sweep gas flow rate | 0 |
| Spray voltage ( lKVl ) | -2.5 |
| Capillary temp. (℃) | 320 |
| Aux gas heater temp(℃) | 30 |
| Resolution | 140000 |
| Polarity | Negtive |
| In-source CID (eV) | 20 |
| Maximum inject time(ms) | 1000 |

**Table S3** Summary of spectral indexes of DOM

|  | SUVA254 | E_2_/E_3_ | BIX | HIX | FIX |
| --- | --- | --- | --- | --- | --- |
| Soil | 1.46±0.12 | 5.85±0.25 | 0.62±0.02 | 0.90±0.08 | 1.91±0.08 |
| OCT | 0.43±0.03 | 5.97±0.36 | 0.84±0.03 | 0.86±0.05 | 1.89±0.05 |
| CCT | 1.14±0.08 | 5.24±0.16 | 0.77±0.01 | 0.90±0.02 | 1.93±0.06 |
| OCL | 1.48±0.11 | 4.91±0.09 | 0.65±0.03 | 0.94±0.07 | 2.01±0.08 |
| CCL | 1.31±0.09 | 5.36±0.23 | 0.65±0.02 | 0.92±0.03 | 1.91±0.04 |

**Table S4** Weighted average (wa) values of the molecular composition of the sample

| 样品名 | Molecular formula number | MW_wa_ | O/C_wa_ | N/C_wa_ | S/C_wa_ | H/C_wa_ | AI_mod. wa_ | DBE_wa_ | NOSC_wa_ | DBE-O_wa_ | DBE/C_wa_ |
| --- | --- | --- | --- | --- | --- | --- | --- | --- | --- | --- | --- |
| Soil | 535±32 | 282.08  ±24.02 | 0.37  ±0.02 | 0.11  ±0.01 | 0.06  ±0.01 | 1.48  ±0.03 | 0.24  ±0.01 | 5.47  ±0.62 | -0.29  ±0.02 | 1.27  ±0.08 | 0.41  ±0.03 |
| OCT | 478±21 | 270.07  ±28.13 | 0.33  ±0.05 | 0.12  ±0.02 | 0.09  ±0.02 | 1.22  ±0.02 | 0.38  ±0.02 | 6.40  ±0.57 | -0.03  ±0.01 | 2.59  ±0.13 | 0.54  ±0.05 |
| CCT | 465±28 | 266.78  ±18.37 | 0.37  ±0.03 | 0.12  ±0.01 | 0.09  ±0.03 | 1.21  ±0.01 | 0.37  ±0.02 | 6.32  ±0.48 | 0.06  ±0.01 | 2.28  ±0.12 | 0.55  ±0.07 |
| OCL | 504±25 | 309.58  ±33.56 | 0.27  ±0.02 | 0.05  ±0.03 | 0.04  ±0.01 | 1.48  ±0.02 | 0.24  ±0.01 | 5.95  ±0.51 | -0.72  ±0.02 | 2.11  ±0.09 | 0.35  ±0.04 |
| CCL | 750±36 | 278.52  ±23.74 | 0.36  ±0.03 | 0.09  ±0.02 | 0.05  ±0.01 | 1.46  ±0.02 | 0.25  ±0.01 | 5.55  ±0.63 | -0.38  ±0.01 | 1.50  ±0.05 | 0.40  ±0.03 |

**Table S5** Data of microbial community richness and diversity

|  | OTUs | Chao1 | Shannon |
| --- | --- | --- | --- |
| Soil-0 | 2010±198 | 2011.52±46.74 | 3.78±0.07 |
| Soil-OC45 | 2438±226 | 2438.66±68.56 | 6.82±0.13 |
| Soil-CC45 | 2781±251 | 2781.67±73.38 | 7.91±0.28 |
| Soil-OC120 | 2997±278 | 2997.43±97.21 | 8.63±0.56 |
| Soil-CC120 | 3070±276 | 3070.38±109.36 | 8.71±0.95 |

**Figure S1** Ultraviolet-visible spectroscopy of DOM.

**Figure S2** Fluorescence spectrum of various DOM.

**Figure S3** (a) Dilution curve of microbial community samples; (b) PCoA figure calculated based on Bray-Curtis sample distance information.
